# Disposition, Metabolism and Histone Deacetylase and Acetyltransferase Inhibition Activity of Tetrahydrocurcumin and Other Curcuminoids

**DOI:** 10.3390/pharmaceutics9040045

**Published:** 2017-10-12

**Authors:** Júlia T. Novaes, Ryan Lillico, Casey L. Sayre, Kalyanam Nagabhushanam, Muhammed Majeed, Yufei Chen, Emmanuel A. Ho, Ana Luísa de P. Oliveira, Stephanie E. Martinez, Samaa Alrushaid, Neal M. Davies, Ted M. Lakowski

**Affiliations:** 1 The Rady Faculty of Health Sciences, College of Pharmacy, Pharmaceutical Analysis Laboratory, University of Manitoba, Winnipeg, MB R3E 0T5, Canada; juliatnovaes@gmail.com (J.T.N.); umLillic@myumanitoba.ca (R.L.); umche355@myumanitoba.ca (Y.C.); analuisapoliv@gmail.com (A.L.d.P.O.); umalrush@myumanitoba.ca (S.A.); 2 College of Pharmacy, Roseman University of Health Sciences, South Jordan, UT 84096, USA; csayre@roseman.edu; 3 Sabinsa Corporation, 20 Lake Drive, East Windsor, NJ 08520, USA; kalyanam@sabinsa.com (K.N.); mmjd52@hotmail.com (M.M.); 4 Faculty of Science, School of Pharmacy, University of Waterloo, Kitchener, ON N2G 1C5, Canada; emmanuel.ho@uwaterloo.ca; 5 Department of Veterinary Clinical Sciences, College of Veterinary Medicine, Washington State University, Pullman, WA 99164-6610, USA; smartinez@vetmed.wsu.edu; 6 Faculty of Pharmacy and Pharmaceutical Sciences, University of Alberta, Edmonton, AB T6G 2R3, Canada

**Keywords:** curcumin, tetrahydrocurcumin, calebin-A, UHPLC–MS/MS, pharmacokinetics, antioxidant, anti-inflammatory, anticancer, HDAC, HAT

## Abstract

Tetrahydrocurcumin (THC), curcumin and calebin-A are curcuminoids found in turmeric (*Curcuma longa*). Curcuminoids have been established to have a variety of pharmacological activities and are used as natural health supplements. The purpose of this study was to identify the metabolism, excretion, antioxidant, anti-inflammatory and anticancer properties of these curcuminoids and to determine disposition of THC in rats after oral administration. We developed a UHPLC–MS/MS assay for THC in rat serum and urine. THC shows multiple redistribution phases with corresponding increases in urinary excretion rate. In-vitro antioxidant activity, histone deacetylase (HDAC) activity, histone acetyltransferase (HAT) activity and anti-inflammatory inhibitory activity were examined using commercial assay kits. Anticancer activity was determined in Sup-T1 lymphoma cells. Our results indicate THC was poorly absorbed after oral administration and primarily excreted via non-renal routes. All curcuminoids exhibited multiple pharmacological effects in vitro, including potent antioxidant activity as well as inhibition of CYP2C9, CYP3A4 and lipoxygenase activity without affecting the release of TNF-α. Unlike curcumin and calebin-A, THC did not inhibit HDAC1 and PCAF and displayed a weaker growth inhibition activity against Sup-T1 cells. We show evidence for the first time that curcumin and calebin-A inhibit HAT and PCAF, possibly through a Michael-addition mechanism.

## 1. Introduction

**Figure 1:**
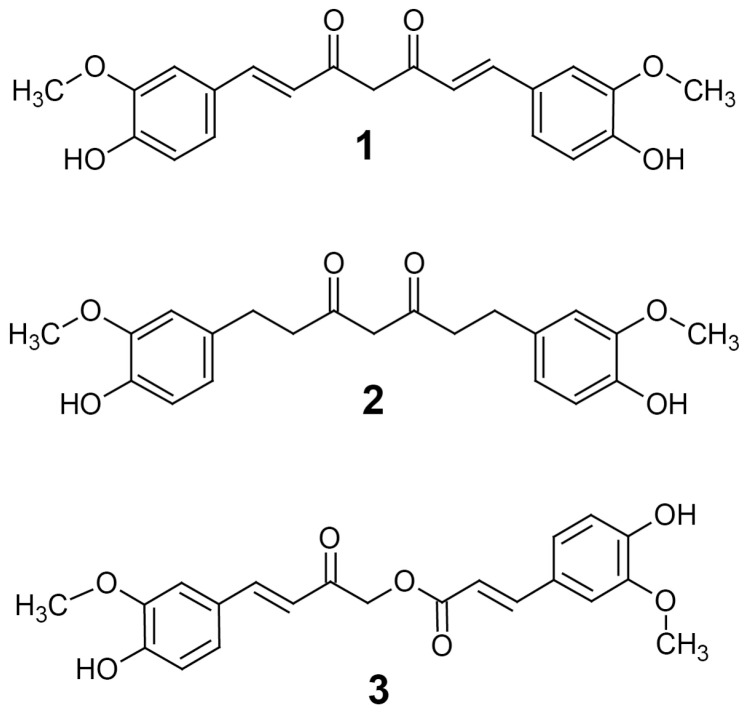
The structures of curcuminoids curcumin (**1**), tetrahydrocurcumin (THC) (**2**) and calebin-A (**3**). Both THC and curcumin are shown in the keto tautomer.

Turmeric from *Curcuma longa* is a popular spice known to contain curcuminoids and has been used traditionally as a natural health product [1]. There are many compounds of the curcuminoid family that are thought to produce its effects, the most studied being curcumin (**1**) (Figure 1) [2]. Curcumin is a deep yellow, poorly water-soluble substance that has been claimed to exhibit a variety of effects including antioxidant, anti-inflammatory, antiviral, antifungal, antibacterial, anticancer, antidiabetic and neuroprotective properties [3]. The effects elicited by curcumin are thought to be partly a result of its activity inhibiting the histone lysine acetyltransferases (HATs) p300/CBP [4] as well as histone deacetylases (HDACs) such as HDAC1 [5]. Inhibition of enzymes that catalyze lysine acetylation are known to have a variety of effects on gene expression and such activity confers a potential epigenetic mechanism to curcumin that has led to its exploration as an anticancer medication. Indeed, several HDAC inhibitors are already in use alone or in combination for the treatment of cancer and several groups have thought to exploit curcumin for its HDAC inhibition activity. This activity has been shown to suppress some DNA damage repair pathways and may be used to increase the effectiveness of existing cancer treatments [6]. Despite its poor bioavailability, curcumin has even been shown to suppress medulloblastoma growth in vivo through its HDAC inhibition activity [7]. Inhibition of HAT is currently being explored as a new class of cancer treatment, but despite promising results, no HAT inhibitors are currently approved; therefore, the anticancer potential of the HAT inhibition of curcumin has not been explored as much as its HDAC inhibition activity [8]. Curcumin is thought to exhibit its HAT inhibition activity by a Michael-addition reaction, alkylating the active-site cysteines of HAT [9].

Another important consequence of inhibiting HAT activity is the repression of genes important in the inflammatory response. For example, some HAT inhibitors have been shown to inhibit the NF-κB pathway [10,11]. Recently, HAT inhibitors having a similar mechanism of inhibition to curcumin [9] have been shown to reduce expression of COX2, yielding anti-inflammatory and analgesic effects [12]. Although a great deal of attention has been paid to curcumin, there are many other curcuminoid-type compounds in turmeric that have potential pharmacological activities, such as tetrahydrocurcumin (THC) (**2**) and calebin-A (**3**) (Figure 1) [2].

Calebin-A has been studied for its antioxidant, anti-inflammatory and anticancer effects that have been demonstrated in vitro by inhibition of cyclooxygenase and lipoxygenase; it may also have cytochrome P450 inhibitory activity, as it appears to interact with CYP2D6 and CYP1A2 [2]. It has also been shown to inhibit dipeptidyl peptidase-4 (DPP-4), which is a membrane glycoprotein and serine exopeptidase that is implicated in type 2 diabetes mellitus through its activity inhibiting incretin release, including GLP-1 [13]. Inhibitors of DPP-4, such as gliptins, are currently used clinically as hypoglycemic agents. Calebin-A has potential as an adjuvant for cancer therapy, increasing the effectiveness of currently used cancer treatments, as it has been shown to directly inhibit P-glycoprotein and has been co-administered with vincristine to treat multidrug-resistant human gastric cancer cells. Furthermore, calebin-A also appears to modulate the mitogen-activated protein-kinase pathway. These data suggest the calebin-A may be an effective cancer treatment [1], however, a major drawback is that it has an extremely low oral bioavailability, much like curcumin [2].

THC is another curcuminoid found in turmeric that is produced by the reduction of curcumin [14]. It can be synthesized in the laboratory by hydrogenation of curcumin, yielding a colorless substance. THC can also be produced in vivo through Phase I metabolism in the liver by hepatic reductases, and as a result, after oral administration it is found as free THC and conjugated as glucuronides. Like curcumin and calebin-A, THC is also thought to have several biological activities; among the most studied are its antioxidant properties. In fact, the antioxidant role of THC has been studied as a potential preventative treatment for cardiovascular disease by reducing the formation of atherosclerotic lesions. THC has also been studied for its anti-inflammatory activity, both as a treatment for arthritis and cancer [14]. Like calebin-A and curcumin, THC is an inhibitor of P-glycoprotein, and because of this, THC has been explored as a potential way to overcome multidrug resistance in cancer [15].

THC has a number of attractive properties not shared with curcumin that may make it superior. For example, THC is significantly more stable under physiological pH and temperature conditions than curcumin [16]. Moreover, it is slightly more soluble in water, making it much easier to formulate into an acceptable dosage form than curcumin which, as stated above, has had longstanding issues with its near-insolubility in water and a number of other common solvents.

Currently, there are few studies in the literature about curcuminoids, apart from curcumin. Considering the depth of study of curcumin as a treatment for cancer among other diseases, the fact that curcumin is metabolized to THC, and the fact that THC has therapeutic potential in its own right, it is necessary to describe the pharmacological effects and pharmacokinetic characteristics of THC. In this study we developed a bioanalytical assay to quantify THC in urine and serum using ultra-high-performance liquid chromatography–mass spectrometry (UHPLC–MS/MS) to characterize its pharmacokinetics in rats after oral (PO) administration. We also identify the HDAC and HAT activities, as well as the antioxidant, anti-inflammatory and cell viability activities of THC compared to curcumin and calebin-A. As far as we know, this is the first study to dose rats with THC and measure serum and urine concentrations of THC.

## 2. Materials and Methods

### 2.1. Chemicals and Reagents

Tetrahydrocurcumin, curcumin and calebin-A were provided by Sabinsa Corporation^®^ (Piscataway, NJ, USA). Sulfaphenazole, ketoconazole, α-naphthoflavone, quinidine, DMSO, PEG-400 and β-glucuronidase type IX A (β-glucuronidase) were purchased from Sigma-Aldrich (St. Louis, MO, USA). Analytical-grade formic acid and HPLC-grade acetonitrile were purchased from Fisher Scientific. Ultrapure water from a Milli-Q^®^ system (Millipore, Billerica, MA, USA) was used for mobile phase. The antioxidant activity kit, cyclooxygenase-1 and -2 inhibitor screening kits, lipoxygenase inhibitor screening kit, HDAC1 and PCAF inhibitor screening kits were purchased from Cayman Chemical Company (Ann Arbor, MI, USA). Vivid^®^ CYP2C9 green screening kit, Vivid^®^ CYP3A4 green screening kit, Vivid^®^ CYP1A2 blue screening kit and Vivid^®^ CYP2D6 blue screening kit were bought from Life Technologies™ (Carlsbad, CA, USA). Human CXCL9/MIG, Human TNF-α, Human IFN-γ and Human CXCL10/IP-10 DuoSet^®^ ELISA kits, solutions and reagents were purchased from R & D Systems^®^ (Minneapolis, MN, USA). CellTiter 96^®^ AQueous One Solution Cell Proliferation Assay (MTS) was purchased from Promega (Madison, WI, USA). Heat-inactivated fetal bovine serum was purchased from Gibco^®^ by Life Technologies™ and Roswell Park Memorial Institute (RPMI) 1640 medium and Penicillin Streptomycin solution were purchased from Corning.

### 2.2. UHPLC–MS/MS Analysis and Conditions

THC was analyzed using the Shimadzu Nexera UHPLC coupled to a Shimadzu LCMS 8040 triple quadrupole mass spectrometer (Shimadzu, Kyoto, Japan). Chromatography was performed using a Waters Acquity UPLC BEH C18 column under isocratic conditions with 45% aqueous acetonitrile in 0.1% formic acid at a flow rate of 0.4 mL/min. THC and the internal standard (curcumin) were measured using Multiple Reaction Monitoring (MRM) in positive mode using the transitions *m*/*z* 373.3 > 137.1 and *m*/*z* 369.3 > 177.1, respectively. Data analysis was performed with the LabSolutions software version 5.72 (Shimadzu, Kyoto, Japan).

### 2.3. Animals

Surgically-modified, exposed jugular vein-catheterized (polyurethane-silastic blended catheter), adult male CD Sprague-Dawley rats (250–300 g) were purchased from Charles River Laboratories (St. Constant, QC, Canada). Rats received free access to food (Purina Rat Chow 5001) and water in the animal facility for at least 3 days before use. Rats were housed in temperature-controlled rooms with a 12 h light/dark cycle. Animal ethics approval was obtained from University of Manitoba Office of Research Ethics and Compliance (protocol 11-064) and a minimal number of rats were utilized to answer the hypothesis tested.

### 2.4. Pharmacokinetic Study

THC was provided as a dry powder from Sabinsa Corporation^®^ and this was dissolved in a mixture of DMSO 2% and 98% PEG-400. Each rat was placed in a separate metabolic cage and fasted for 12 h prior to dosing with free access to water. On the day of experiment, the animals (*N* = 3) received a single dose of THC by oral gavage (500 mg/kg) in a volume not exceeding 1 mL. Animals had free access to water pre- and post-dosing, and food (Purina Rat Chow 5001) was provided 2 hours post-dosing. A series of blood samples (0.3 mL) were collected at 0, 15 and 30 min, and 1, 2, 4, 6, 12, 24, 48 and 72 h post-dose. At 72 h after administration, the animals were euthanized and exsanguinated. Immediately after each blood collection time point (except the terminal point), the cannula was flushed with 0.3 mL of 0.9% saline to replenish the collected blood volume. The dead volume of the cannula was replaced with sterile heparin/50% dextrose catheter lock solution (SAI Infusion Technologies, Strategic Applications, Lake Villa, IL, USA) to maintain the patency of the cannula as advised in the technical sheet supplied with the animals from Charles River. Following centrifugation of blood samples at 15,000 rpm for 5 min, serum was collected and placed into 2 mL tubes at −20 °C until further analysis. Urine samples were collected at 0, 2, 6, 12, 24, 48 and 72 h post-dose and placed in 15 mL tubes. The exact urine volume of each sample was recorded then stored at −20 °C until further analysis.

### 2.5. Standard Solutions and Standard Curve Preparation

Stock solutions of THC and curcumin were prepared in acetonitrile and calibration standards were prepared from these stocks in blank urine and serum into a series of concentrations: 0.005, 0.01, 0.05, 0.1, 0.5, 1.0, 5.0 and 10.0 µg/mL. Briefly, 100 µL of serum or urine, 50 µL of internal standard (curcumin 1 µg/mL) and the corresponding concentration of THC stock solution were combined and vortexed in a 2 mL microtube. Ice-cold acetonitrile (1 mL) was added to precipitate serum proteins from solution and the same was done in urine for consistency. These solutions were vortexed, centrifuged and the supernatants were collected and evaporated to dryness by a stream of nitrogen gas. The pellet was reconstituted with 50 μL of 45% aqueous acetonitrile (ACN) with 0.1% formic acid (F.A.) and injected into the UHPLC–MS/MS. The accuracy was measured from three standard curves on three separate days and precision measured with 4 quality control (QC) samples at the low, mid and high range of the curve. The accuracy and precision of the assay were 89.2 ± 10% and 95.1 ± 4.9%, respectively.

### 2.6. Serum and Urine Sample Preparation

Serum and urine samples (100 μL) were treated with 20 μL of 500 U/mL β-glucuronidase, vortexed and incubated at 37 °C for 2 h to release any glucuronide conjugates. 50 µL of internal standard (curcumin 1 µg/mL) was added, vortexed and rapidly, 1 mL of cold acetonitrile was added to precipitate the proteins present in both samples. Urine and serum samples were vortexed and centrifuged at 15,000 rpm for 5 min. The supernatants were collected and evaporated to dryness by nitrogen gas. Samples were reconstituted with 50 μL of mobile phase (45% ACN, 0.1% F.A.) and vortexed. The samples were transferred to HPLC vials and 10 μL was injected into the UHPLC system for each sample.

### 2.7. Cytochrome P450 Inhibition Determination

CYP2C9, CYP3A4, CYP1A2 and CYP2D6 assays were performed to assess metabolism and inhibition of human P450 isozymes involved using Vivid^®^ CYP450 screening kits and CYP450 BACULOSOMES^®^ Plus Reagents. CYP450 BACULOSOMES^®^ Plus Reagents express only one CYP450 enzyme, preventing metabolism by other CYP450s. For CYP2C9, the substrate was BOMF and the positive control inhibitor sulfaphenazole; for CYP3A4, DBOMF was the substrate and ketoconazole the positive control inhibitor; for CYP1A2, the substrate was EOMCC and the positive control inhibitor naphthoflavone; for CYP2D6, the substrate was EOMCC and the positive control inhibitor quinidine. The assays were performed using the excitation and emission wavelengths recommended by the manufacturer. To run the assay, 50 μL of Master Pre-Mix was added with 40 μL of THC, curcumin and calebin-A, at 0.01, 0.1, 1.0, 10.0, 50.0 and 100.0 μM dissolved in DMSO. The plate was incubated for 10 min at room temperature (25 ± 1 °C) on a plate shaker. Then, 10 μL of Vivid^®^ Substrate was added to each well to start the reaction. Less than 2 min after the reaction had started, the measurement of fluorescence was performed using the Synergy HT multi-well plate reader and Gen5 data analysis software (Biotek Instruments Inc., Winooski, VT, USA). The assay was done in triplicate. The reading was performed at 1 min intervals for 60 min. Inhibition was calculated as (1 − (X − B/A − B)) × 100%, where X was the average of compound fluorescence, A was the average of solvent control and B was the average of inhibition in the presence of positive inhibitor control. The percent inhibition was calculated relative to a fixed concentration of the positive control. For more information of assay protocols, please refer to the instructions for the kits (Vivid^®^ CYP2C9 Green Screening Kit—Cat. no. P2860; Vivid^®^ CYP3A4 Green Screening Kit—Cat. No. P2857; Vivid^®^ CYP1A2 Blue Screening Kit—Cat. No. P2863 and Vivid^®^ CYP2D6 Blue Screening Kit—Cat. No. P2972 from Life Technologies).

### 2.8. Antioxidant Capacity Determination

Total antioxidant capacity of THC, curcumin and calebin-A was measured using the antioxidant assay kit (Cayman Chemical, Ann Arbor, MI, USA) following the standard protocol from the manufacturer. The assay measures the inhibition of oxidation of 2,2’-azino-bis(3-ethylbenzothiazoline-6-sulphonic acid) (ABTS) by metmyoglobin. THC, curcumin and calebin-A were prepared in DMSO for concentrations of 1, 5, 10, 50 and 100 μg/mL. The antioxidant capacity of each curcuminoid was expressed as Trolox equivalents. The assay was performed in triplicate and absorbance at 750 nm was measured using the Synergy HT plate reader (Biotek Instruments Inc., Winooski, VT, USA). For more information regarding the assay protocol, please refer to the instructions for the kit (Antioxidant Assay kit from Cayman Chemical—Cat. No. 709091).

### 2.9. Cyclooxygenase Inhibition Determination

Cyclooxygenase is a bifunctional enzyme exhibiting both COX and peroxidase activities. This assay was used to measure the inhibition of COX-1 and -2 of THC, curcumin and calebin-A. Concentrations of 1, 10 and 250 μg/mL of THC, curcumin and calebin-A were prepared using DMSO. The assay was performed in quadruplet using a COX Inhibitor Screening Assay Kit from Cayman Chemical (Cat. No. 560131) according to the manufacturer’s instructions. The measurement of absorbance was performed at 415 nm within 10 min at room temperature using the Synergy HT multi-well plate reader and Gen5 data analysis software (Biotek Instruments Inc., Winooski, VT, USA).

### 2.10. Lipoxygenase Inhibition

We detected the lipoxygenase activity of the curcuminoids using a Lipoxygenase Inhibitor Screening Assay Kit from Cayman Chemical (Ann Arbor, MI, USA, Cat. No. 760700a) using linoleic acid as the substrate according to the manufacturer’s recommended instructions. Briefly, concentrations of 1, 10 and 250 μg/mL of THC, curcumin and calebin-A were prepared in DMSO and 10 μL of each were combined with 90 μL of 15-lipoxygenase derived from soybean. 10 μL of linoleic acid was added to start the reaction. The plate was covered and placed on a shaker for 5 min. Then, 100 μL of chromogen was added. The plate was covered and placed on a shaker for 5 min again. The assay was performed in triplicate. The absorbance was measured at 500 nm using the Synergy HT multi-well plate reader and Gen5 data analysis software (Biotek Instruments Inc., Winooski, VT, USA). For additional information of assay protocol, please refer to the instructions for the kit.

### 2.11. Cellular Growth Inhibition Assay (MTS)

The CellTiter 96^®^ AQueous One Solution Cell Proliferation Assay (MTS, Promega, Madison, WI, USA) was used to measure proliferation inhibition of cultured cells. Briefly, Sup-T1 cells were cultured in RPMI 1640 supplemented with 10% FBS and 1% penicillin/streptomycin at 37 °C and 5% CO_2_. 2 × 10^5^ cells/mL were seeded in each well and THC, curcumin and calebin-A, at 0.1, 0.5, 1.0, 5.0, 10.0, 50.0 and 100.0 μM dissolved in DMSO, were added to their respective wells and incubated for 24, 48 and 72 h. The MTS reagent was added and incubated for 4 h. Absorbance was recorded at 490 nm in Synergy HT multi-well plate reader and Gen5 data analysis software (Biotek Instruments Inc., Winooski, VT, USA). Sup-T1 cells were used in this study because curcuminoids are thought in part to work via an HDAC inhibitor mechanism and as some HDACs are approved for some T-cell lymphomas, we used Sup-T1 cells because they are a T-cell lymphoma cell line that is established. To the best of our knowledge no other study has measured the effects of THC on Sup-T1 cells.

### 2.12. Cellular Inflammatory Cytokine Release Assay (TNF-α)

Sup-T1 cells were prepared and seeded as above and separately treated with THC, curcumin and calebin-A at 5, 10, 50 and 100 μM. Cell culture supernatant was collected and used to determine released TNF-α using the Human TNF-alpha DuoSet ELISA kit (DY210, R & D systems, Minneapolis, MN, USA) following the standard manufacturer’s protocol, optimized using recombinant TNF-α. The optical density was determined in Synergy HT multi-well plate reader and Gen5 data analysis software (Biotek Instruments Inc., Winooski, VT, USA). The data were analyzed using SigmaPlot 12.2 (Systat Software Inc., San Jose, CA, USA).

### 2.13. HDAC1 and PCAF Inhibition Assays

Curcuminoids were tested for direct HDAC1 and PCAF inhibitory activity using the fluorescent-based HDAC1 or PCAF inhibitor screening assay kit ((HDAC1) 10011564, (PCAF) 10006515, Cayman, Ann Arbor, MI, USA) according to manufacturer’s instructions. THC, curcumin and calebin-A, at 0.1, 1.0, 10.0, 50.0, 100.0 and 250.0 μg/mL dissolved in DMSO, were prepared and screened. Fluorescence was measured for both assays using excitation/emission wavelengths of 340/460 nm using Synergy HT multi-well plate reader and Gen5 data analysis software (Biotek Instruments Inc., Winooski, VT, USA).

Data for the PCAF and HDAC1 inhibition and viability assays were used to derive percent changes based on no treatment controls. Each of three data sets were then fit to a 4-parameter logistic regression using SigmaPlot 12.2 to derive IC_50_ values, which were then used to determine the mean and SD.

## 3. Results

### 3.1. Chromatography

**Figure 2:**
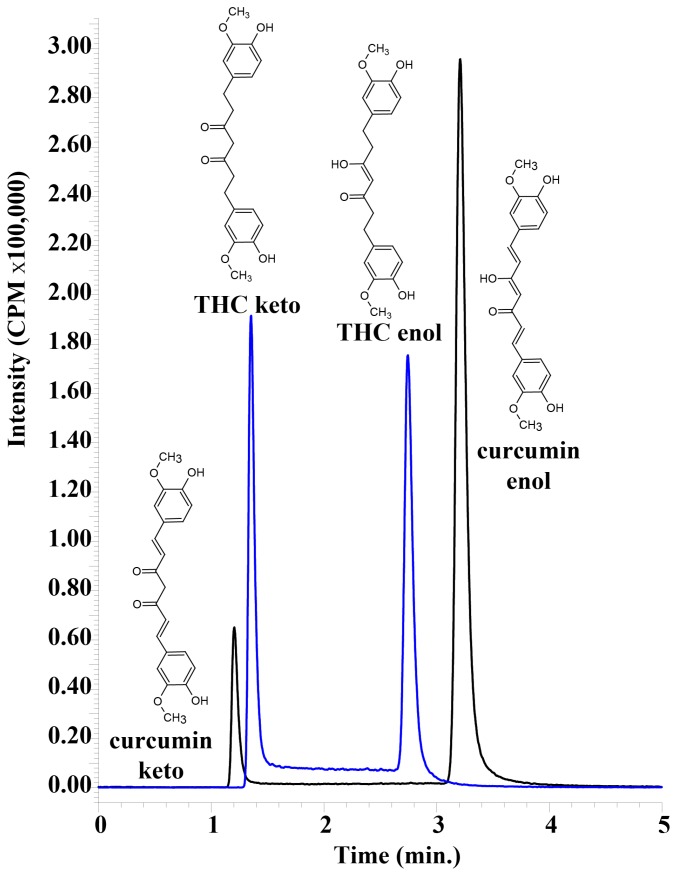
A representative chromatogram of curcumin and tetrahydrocurcumin (THC) Shown are the enol and keto forms of curcumin and THC using the LC–MS/MS assay. Both compounds are at a concentration of 1 μg/mL dissolved in acetonitrile.

The central 3,5-dione structure of THC and curcumin allows for keto–enol tautomerism. We optimized chromatographic conditions for both tautomers of THC to be resolved easily as the keto and enol forms that had a retention time of 1.4 min and 2.7 min, respectively. Similarly, the keto and enol tautomers of curcumin were easily resolved, having retention times of 1.2 min and 3.2 min, respectively (Figure 2). The enol and keto forms of each compound have the same molecular weight and fragmentation spectra, so the mass spectrometer on its own is incapable of differentiating the two tautomers of each analyte. We reconciled the retention times of each tautomer based on the previous studies which showed that curcumin is primarily in the enol form in solutions of ~50% acetonitrile, which is very similar to the conditions we used [17]. Corroborating this, other similar studies have shown that in aqueous solutions with increasing amounts of alcohol the enol tautomer predominates [18]. We defined the larger peak with the longer retention time to be the enol tautomer for curcumin, as has been observed by others under similar conditions [17], and used the same relative retention to identify the tautomers of THC (Figure 2). For THC, we found that the two tautomers were in roughly equal proportion under these chromatographic conditions, while curcumin appeared to favour the enol tautomer by a 5:1 ratio. The sum of the peak areas for both tautomers was used to calculate the concentrations in the samples. Blank plasma samples showed that there were no interfering peaks that co-eluted with THC and curcumin (data not shown).

### 3.2. Pharmacokinetics

**Figure 3:**
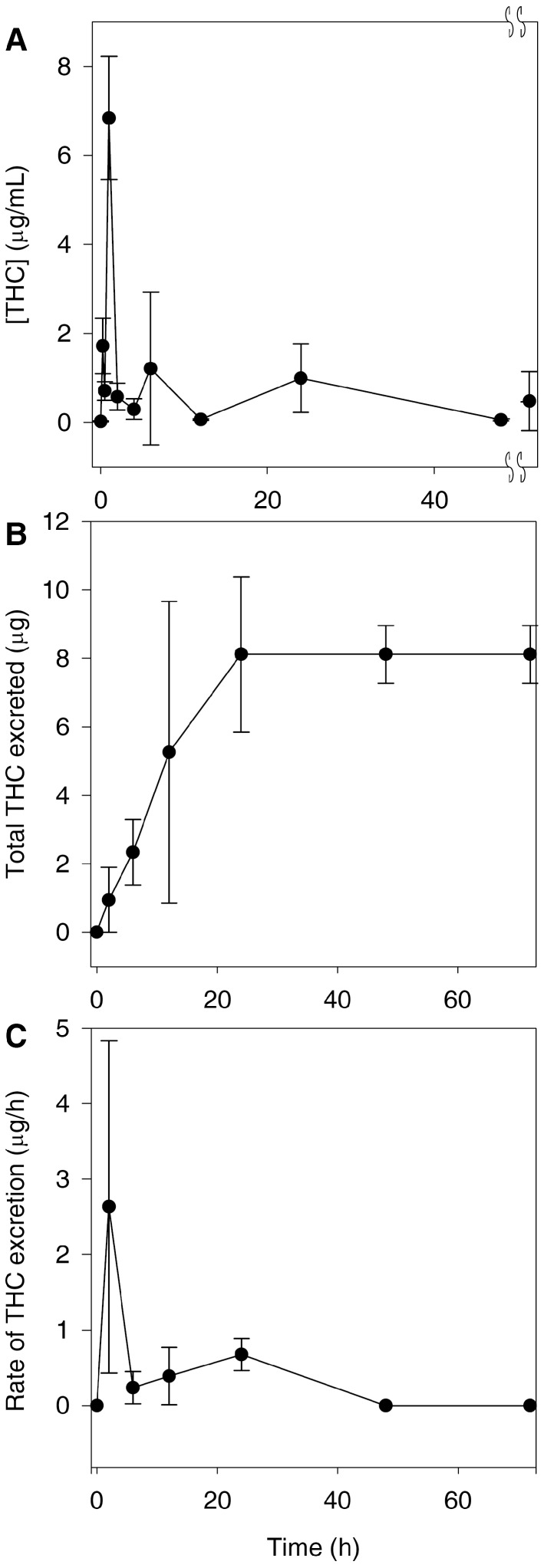
The pharmacokinetics of THC after a 500 mg/kg oral dose. Depicted is the serum disposition of THC in μg/mL (**A**) (the double S-marks represent a break in the Time axis from 49 to 71 h and the final time point is 72 h); the total amount of THC excreted in the urine in μg after oral administration (**B**); and the rate of urinary excretion of THC in μg/mL (**C**). All values represent a mean of 3 with standard deviation.

The serum THC concentration versus time curve shows that more than one absorption and distribution phase was present. Initially, a rapid absorption phase with an average *T*_max_ of 6.8 μg/mL at 1 h was observed, followed by a short elimination phase. This was followed by two redistributions with two smaller THC maxima at 6 and 24 h (Figure 3A). Both redistribution phases had similar maxima of about 1 μg/mL (Figure 3A). The total amount of THC excreted unchanged in urine was up to 8 μg at 24 h (Figure 3B). Coinciding with the first short elimination phase, the rate of urinary excretion reached a maximum of just under 3 μg/h at 2 h. In addition, an increase in excretion rate can be observed at 12 after the second distribution. Finally, a second excretion rate maximum coincided with the final distribution phase at 24 h.

### 3.3. Cytochrome P450 Inhibition

**Figure 4:**
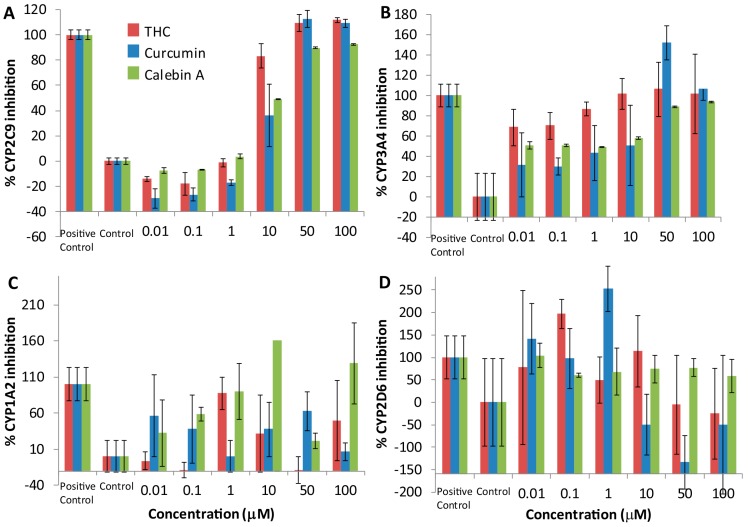
The inhibition of selected common drug-metabolizing enzymes by curcuminoids. Displayed are graphs showing the inhibition of CYP 2C9 (**A**); CYP3A4 (**B**); CYP1A2 (**C**); and CYP 2D6 (**D**) by THC, curcumin and calebin-A with solvent and positive controls using commercial assay kits.

The three curcuminoids were tested for their ability to inhibit CYP 2C9, 3A4, 1A2 and 2D6. THC, curcumin and calebin-A yielded dose-dependent inhibition of CYP2C9, and to a lesser extent, CYP3A4. All three exhibited maximum inhibition of CYP2C9 (Figure 4A) and CYP3A4 (Figure 4B) at 50 to 100 μM, compared to the positive controls sulphaphenazole and ketoconazole. Curcumin, calebin-A and THC did not show a consistent dose-response inhibition of CYP1A2 (Figure 4C) or CYP2D6 (Figure 4D) over the range of concentrations tested. In some cases, the percent inhibition exceeded 100%; this may be because percent inhibition is calculated relative to a fixed concentration of a positive control inhibitor. In those cases where percent inhibition exceeds 100%, it may be because the curcuminoid being studied is a more effective inhibitor of the CYP in question than the positive control. For Figure 4D, in some cases, curcumin appears to produce negative inhibition or increased activity, but the error associated with these values makes it difficult to determine if this is the case. 

### 3.4. Anti-Inflammatory Activity

**Figure 5:**
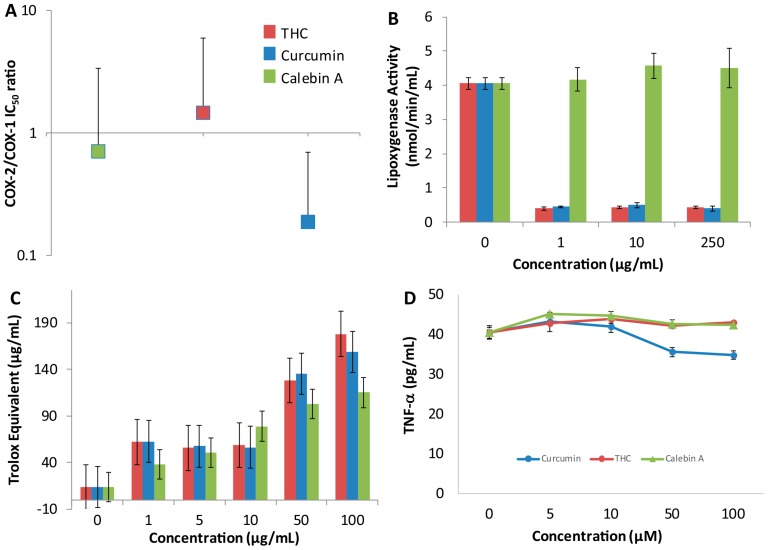
The anti-inflammatory and antioxidant activity of THC and other curcuminoids. The direct effects of curcuminoids on inhibition of cyclooxygenase displayed as the COX-2 to COX-1 IC_50_ ratio (**A**); the inhibition of lipoxygenase by THC and other curcuminoids displayed as lipoxygenase activity in mmol/min/mL at the listed concentrations of curcuminoids (**B**); the antioxidant activity of THC and other curcuminoids as measured by the antioxidant assay kit (Cayman) (**C**); displayed are the TNF-α concentrations produced by the curcuminoids as a measure of inflammatory activity in pg/mL (**D**). Using a one-way ANOVA, the results for 50 and 100 μM levels are statistically significant *p* < 0.001. All values represent a mean of 3 with standard deviation.

The potential anti-inflammatory activity of HAT inhibitors, like curcumin and other curcuminoids, is thought to involve inhibition of expression of pro-inflammatory genes such as the *NF-κB* family and *COX-2*, however, it is still unclear if there is any direct inhibitory activity for curcuminoids against these pro-inflammatory proteins. Therefore, the inhibition of cyclooxygenase and lipoxygenase by THC, curcumin and calebin-A was measured using assay kits described in the methods. For the cyclooxygenase inhibition assay, we measured COX-2 and COX-1 IC_50_ and reported their COX-2/COX-1 ratios. Calebin-A and THC have similar ratios around 1, demonstrating approximately equal propensity to directly inhibit COX-2 and COX-1. Curcumin appears to show higher COX-2 inhibition with a COX-2/COX-1 ratio of 0.19 (Figure 5A). None of the curcuminoids were particularly effective inhibitors of either COX-1 or -2, with curcumin being the most effective against COX-2 with an IC_50_ of ~600 μM (Table 1). We evaluated the potential lipoxygenase inhibitory activity because we found marginal COX inhibition. Calebin-A did not show any activity, but both curcumin and THC inhibited lipoxygenase as low as 1 μM (Figure 5B).

The total antioxidant activity of THC, curcumin and calebin-A was calculated with respect to Trolox as a measure of antioxidant capacity. Both THC and curcumin (and to a lesser extent calebin-A) showed their greatest antioxidant effect at 100 μM (Figure 5C).

In order to fully evaluate the anti-inflammatory activity of the curcuminoids, the concentrations of the pro-inflammatory mediators TNF-α, IFN-γ, MIG and IP-10 were measured using an ELISA assay in Sup-T1 cells treated with increasing concentrations of THC, curcumin and calebin-A. The measurements for IFN-γ, MIG and IP-10 were below detection levels for all curcuminoids. There also appeared to be no dose-response change in the concentration of TNF-α in Sup-T1 cells treated with THC and calebin-A. However, a slight decrease in TNF-α compared to no-treatment control appears to be present at the 50 and 100 μM levels for curcumin (Figure 5D).

### 3.5. Cell Viability

**Figure 6:**
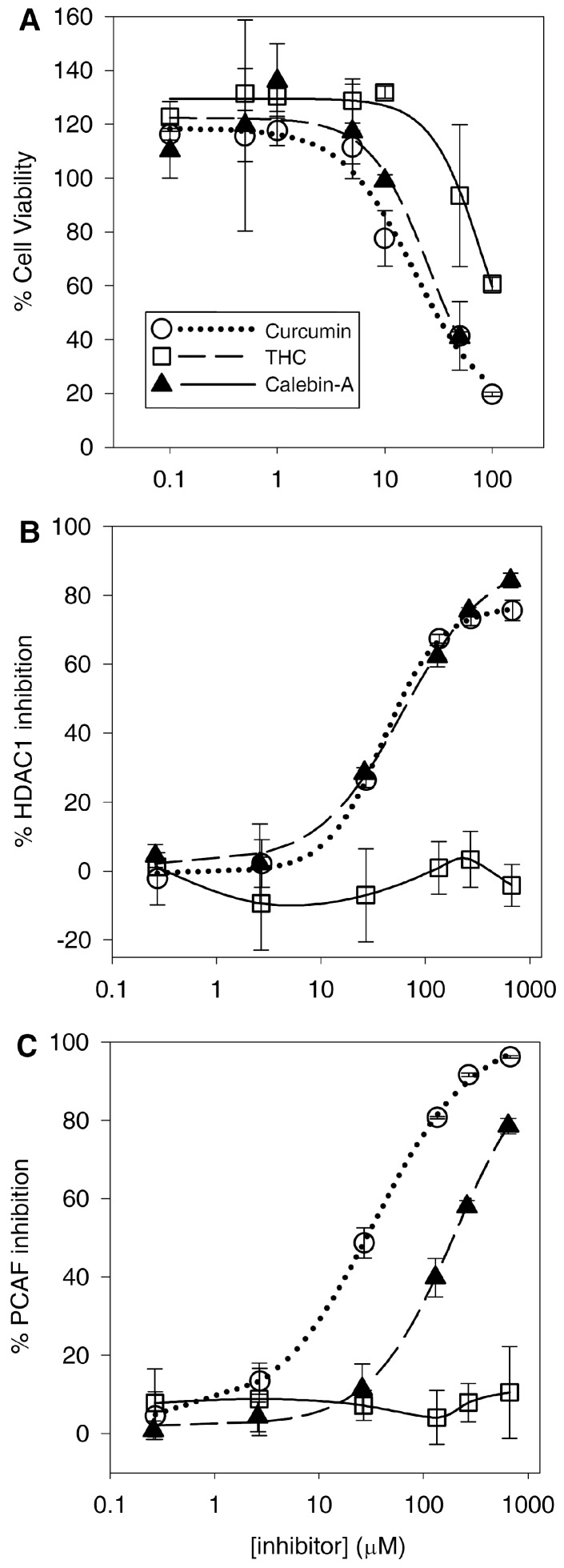
Cell viability and HDAC and HAT inhibition. The viability of Sup-T1 cells with increasing concentrations of the curcuminoids (**A**); the direct inhibition of the HAT PCAF by the curcuminoids as measured by an ELISA assay (**B**); the direct inhibition of the HDAC HDAC1 by the curcuminoids as measured by an ELISA assay (**C**). For each graph the points are the means of three values with an SD.

One potential therapeutic use of curcumin and THC is for the treatment of cancer [3]. It has been shown that curcumin and potentially other curcuminoids are inhibitors of HDAC and HAT. Such compounds that inhibit histone lysine acetylation and deacetylation are already being explored for their cancer treatment potential [9]. The effect of THC, curcumin and calebin-A on cancer cell viability was measured. Sup-T1 cells, T-cell lymphoblastic lymphoma cells, were treated with the curcuminoids to determine their ability to induce growth inhibition using an MTS assay, and the corresponding IC_50_ values were in the mid-to-high micromolar range (Table 2). Curcumin and calebin-A were the most potent, but THC was more than 4-fold less potent than curcumin, and at the most, resulted in a 40% growth inhibition (Figure 6A). 

### 3.6. HDAC1 and HAT Inhibition

Having established the antioxidant, anti-inflammatory and cancer cell growth inhibitory properties of these curcuminoids, their activity against histone acetyltransferases (HAT) and histone deacetylases (HDAC) was evaluated, since it has been previously reported that this may be one of the many possible mechanisms for their anticancer and anti-inflammatory activities. HAT inhibitors are currently being explored as a new class of cancer treatments and have been shown to reduce expression of pro-inflammatory mediators. HDAC inhibitors are already in use alone or in combination to treat various cancers such as cutaneous T-cell lymphoma. We therefore tested the ability of the curcuminoids to inhibit HDAC1 and the HAT PCAF, using a fluorescence-based assay described in the methods. Here, we show curcumin is a potent inhibitor of the HAT PCAF (Figure 6B), an effect that has not been reported previously [4]. Calebin-A is also a PCAF inhibitor but is 6-fold less potent than curcumin (Table 2). Interestingly, THC shows no above-control inhibition of PCAF at any concentration (Figure 6C). Also as expected, curcumin and calebin-A both exhibit HDAC inhibition [5], but with similar potency, and THC shows no inhibitory activity against PCAF or HDAC1 at any concentration (Figure 6 and Table 2).

## 4. Discussion

A sensitive, accurate and reproducible assay was developed for the detection of tetrahydrocurcumin using UHPLC–MS/MS. It was possible to obtain baseline separation between keto and enol forms of THC and curcumin, despite the obvious similarities between the tautomers (Figure 2). Both tautomeric forms of THC and curcumin are present because of the 45% acetonitrile in the mobile phase, and they interconvert on a timescale that results in two peaks on the UHPLC chromatogram that are easily separable [17,18]. Having an alkaline mobile phase would result in only the enol form that would form an enolate, and the two peaks would converge. However, this would necessitate detection in negative MRM mode, which results in a decrease in sensitivity. The acidic mobile phase was important in obtaining the highly sensitive detection of THC and curcumin, but resulted in two separate peaks that needed to be summed in order to calculate total THC and total curcumin. We used UHPLC–MS/MS to unambiguously differentiate curcumin and THC from other serum peaks, and as expected, the plasma samples did not appear to have any other peaks that were erroneously detected as either analyte. 

The choice of curcumin as an internal standard is rational in the sense that its chemical properties and structure are similar to THC. However, as mentioned above, curcumin can be metabolized to THC in the liver, and it is still possible that after plasma samples were spiked with curcumin as the internal standard, a small amount of curcumin could have been metabolized in the plasma. To mitigate this, plasma proteins in the samples were rapidly precipitated with ice-cold acetonitrile, and this would also remove any possible plasma reductases. 

### 4.1. Disposition, Metabolism and Elimination of THC

Preliminary pharmacokinetic studies indicate that un-optimized formulations of THC have poor oral bioavailability, and it appears to only be detectable in serum and urine as glucuronides [19]. In order to detect curcumin and THC by the UHPLC–MS/MS assay, samples were treated with β-glucuronidase in order to proceed with the analysis in serum and urine. Its presence in urine indicates that THC was orally absorbed and was eliminated at least in part through the renal route. However, it has already been long established that curcumin is excreted primarily in the bile as a glucuronide conjugate of THC [20]. More recent evidence suggests that curcumin is excreted into the bile via the active transport ABCC2 drug efflux pump [21]. Therefore, the most likely route of elimination of THC is in the bile as a glucuronide conjugate. This, together with the poor bioavailability of THC, explains why we found low concentrations of THC in the urine (Figure 3B). 

### 4.2. THC, Curcumin and Calebin-A Inhibit CYP2C9 and CYP3A4

Curcuminoids have demonstrated inhibition of many human drug-metabolizing enzymes, especially the Phase I CYP family of oxidases in vitro [22]. We showed that THC, calebin-A and curcumin produced a consistent inhibitory dose-response relationship with CYP2C9 and 3A4, but no such dose-response relationship could be detected with CYP 1A2 and 2D6. These results suggest that THC, curcumin and calebin-A are inhibitors of CYP 2C9 and 3A4. It is not immediately obvious how these curcuminoids inhibit CYP enzymes. Curcuminoids are unlikely to be important as substrates for CYPs, as previous studies have shown that curcumin is reduced to THC by reductases and primarily converted into a THC glucuronide [19,20]. Despite this, a small proportion of each of the curcuminoids may undergo *O*-dealkylation by demethylation of the aromatic methoxy groups, and the occupancy of the enzyme may result in inhibition that is competitive. As has been observed, some curcuminoids have the potential to alkylate enzymes through the α,β-unsaturated carbonyl (Michael acceptor) [9]. This has been the mechanism proposed for curcumin and its activity against p300, however, this cannot be the mechanism of action of CYP inhibition because THC does not have such a Michael acceptor group and yet exhibits CYP inhibition. An alternative mechanism of inhibition may be nucleophilic addition at the enolic carbon (Michael donor) shared between THC and curcumin.

### 4.3. Curcuminoids Inhibit Some Pro-Inflammatory Mediators

None of the curcuminoids were particularly effective inhibitors of either COX-1 or -2, with curcumin being the most effective, yielding a modest IC_50_ of ~600 μM (Table 1). Nevertheless, curcumin showed a slight inhibitory preference for COX-2, suggesting it may have an effect on the activity of COX during acute inflammation. The COX ratios of calebin-A and THC were sufficiently close to 1 and the errors large enough to make it difficult to suggest that either curcuminoid exhibited a preference for either COX enzyme. Furthermore, treatment with THC and calebin-A did not reduce TNF-α release in Sup-T1 cells, and curcumin only had a moderate effect at higher doses. None of the curcuminoids tested altered the concentrations of the cytokines, IFN-γ, MIG and IP-10 in SupT1 cells. Another important group of pro-inflammatory mediators are the leukotrienes, produced by lipoxygenase. Both THC and curcumin inhibited soybean 1,5-lipoxygenase at all doses tested in comparison to a control, while calebin-A showed no such inhibitory activity. As with CYP inhibition, the inhibition of lipoxygenase activity is unclear and cannot be caused by direct Michael addition reaction because THC does not possess a Michael acceptor group, however, both THC and curcumin are very similar in structure, suggesting that this structure may be important for binding to lipoxygenase. Previous studies have used molecular docking approaches to propose potential binding sites for THC on lipoxygenase. These studies suggest that THC scavenges peroxides acting as a redox inhibitor of lipoxygenase, showing mixed inhibition [23]. Other studies confirm that curcumin and THC potently inhibit human lipoxygenase, showing that the inhibition may be clinically significant [24]. The inhibition of lipoxygenase presents the possibility that THC and curcumin may be used as a treatment for inflammatory conditions such as asthma. Lipoxygenase inhibitors such as zileuton are currently in use for treatment of asthma in children where glucocorticoids are less desirable or contraindicated.

Our data showed that THC does not inhibit HDAC or HAT; therefore, its narrower spectrum may make it a superior choice as a lipoxygenase inhibitor anti-inflammatory, because in this case, HDAC and HAT inhibitory activities would represent off-target effects. This is especially significant because we found that THC has a peak concentration of greater than 6 μg/mL (Figure 3A), with concentrations of greater than 1 μg/mL being found up to 20 h later. As we found that THC has potent lipoxygenase inhibition at 1 μg/mL (Figure 5B), this suggests that despite its poor bioavailability, THC reaches concentrations in vivo that can inhibit lipoxygenase. As far as we know, no other group has measured these activities and parameters together and made the suggestion that THC may be used as a lipoxygenase inhibitor to treat asthma and that it may be superior to curcumin. However, we acknowledge that the formulation of THC would need to be dramatically improved or an alternate route of administration chosen. In this particular case, the poor bioavailability of THC may warrant formulation into an intrapulmonary delivery system such as a metered-dose inhaler.

### 4.4. Curcuminoids Are Potent Antioxidants

All curcuminoids tested exhibited antioxidant capacity equivalent to the vitamin E analog antioxidant Trolox in a dose-dependent manner as measured by their free-radical-scavenging activity (ABTS) in the antioxidant assay [16]. All curcuminoids appear to be more potent than Trolox at the 50 and 100 μg/mL levels. However, the data does not suggest there is any difference in antioxidant potency among the curcuminoids. These results suggest that the curcuminoids tested have potential as potent antioxidants if their poor bioavailability can be overcome.

### 4.5. Curcumin and Calebin-A Potently Inhibit Sup-T1 Growth

Consistent with the previously discovered anticancer activity, curcumin induced reductions in the T-cell lymphoblastic lymphoma Sup-T1 cell line. Calebin-A also reduced viability with a similar potency to curcumin (Table 2). However, THC was at least 4-fold less cytotoxic to SupT1 cells compared to curcumin and calebin-A. This difference is likely larger as we were unable to reduce the viability of the Sup-T1 cells below the 50% level over the concentration range used. Therefore, our estimation of the IC_50_ for THC is likely higher than it would be if we were able to reduce the viability below 50%. The Sup-T1 cell line only represents one particular cell line of one type of cancer, and the different curcuminoids may have varying efficacy against different cell and cancer types. For example, THC has been shown to potently inhibit the growth of colorectal cancer cells such as SW480 and HT-29 cells, either alone or in combination [25,26]. In fact, THC has been shown to be more effective than curcumin in colon and other cancers [27,28]. Moreover, the measure of potency for reducing cell viability may not be an accurate reflection of the clinical usefulness of any drug for cancer treatment, as animal cancer models (though outside of the scope for this work) are usually more informative. 

### 4.6. Curcumin and Calebin-A Inhibit PCAF and HDAC1

Consistent with previous findings, curcumin inhibits HDAC and HAT activity. Although previous studies have shown that curcumin binds to and inhibits the HAT activity of p300/CBP [4], as far as we know, this is the first study to show that curcumin and calebin-A inhibit PCAF. These results are not unexpected, as PCAF binds to p300/CBP and all three proteins have HAT activity, albeit with slightly different substrate specificities [29]. The suggested mechanism for inhibition of HAT activity by curcumin is alkylation of the HAT via the Michael acceptor on curcumin. This is thought to happen through HAT active-site cysteine residues [9]. Given this, it is not surprising that calebin-A also inhibits HAT, because it has a similar Michael acceptor group (Figure 1). Previous groups have identified calebin-A as having anticancer activity against a multidrug-resistant human gastric cancer line through its activity in modulating the expression of the MAP kinase family, but the mechanism of this activity was not determined directly [1]. This was recently corroborated by the finding that calebin-A inhibits phosphorylation by suppressing NF-κB signaling activation associated with osteoporosis [30]. This result is important because NF-κB is modulated through the acetylation activity of p300/CBP [31]. Here, for the first time, we can justify the activities against MAPK/NF-κB and the resulting anticancer effects of calebin-A through its direct effect inhibiting PCAF. Furthermore, calebin-A shows similar potency inhibiting HDAC1 compared to curcumin (Figure 6B), which may contribute to their anticancer activities and may be attributed to the presence of the Michael acceptor moiety on each compound. Therefore, the lack of both HDAC1 and PCAF inhibition by THC may be attributed to its lack of double bonds, which form the Michael acceptor groups present in curcumin and calebin-A (Figure 1). The observed absence of HDAC/PCAF inhibition by THC is not entirely surprising as it has long been suspected to have a different anticancer mechanism than curcumin [3]. Several studies have shown that THC is a potent antioxidant, and this study is in agreement with these findings [32,33,34]. Moreover, the finding that THC reduces lipoxygenase activity adds a potential anti-inflammatory mechanism that, in conjunction with its antioxidant activity, may explain the anticancer activity of THC. 

Our results show that curcumin is 6-fold more potent with respect to PCAF inhibition than calebin-A, but both curcuminoids have similar IC_50_ values for inhibition of HDAC1. It is not clear why this might be the case, as both compounds are very similar. It is unlikely that enzymatic or non-enzymatic hydrolysis of the ester of calebin-A might affect its potency, as both compounds have similar potency against HDAC1 under similar conditions to the HAT assay. However, this ester is almost certainly a route of metabolism for calebin-A—that is not present in curcumin or THC—that may affect its in-vivo activity.

## Figures and Tables

**Table 1:** The potency of curcuminoids against cyclooxygenases.

Curcuminoid	COX-1 IC_50_ (μM)	COX-2 IC_50_ (μM)
THC	918 ± 1300	1348 ± 3641
Curcumin	3392 ± 8982	635 ± 367
Calebin-A	1069 ± 490	784 ± 2200

**Table 2:** The potency of curcuminoids on Sup-T1 growth inhibition and HDAC1/PCAF inhibition.

Curcuminoid	Viability IC_50_ (μM)	HDAC1 IC_50_ (μM)	PCAF IC_50_ (μM)
THC	82 ± 116	NA	NA
Curcumin	19.0 ± 11	39.8 ± 3.2	33.1 ± 2.8
Calebin-A	26.2 ± 17	61.3 ± 11	200.0 ± 31
